# Wirelessly powered motor operation in dynamic scenarios using non-Hermitian parity-time symmetry

**DOI:** 10.1038/s41598-023-47842-x

**Published:** 2023-12-06

**Authors:** Shrinathan Esaki Muthu Pandara Kone, Kenichi Yatsugi, Hideo Iizuka

**Affiliations:** Toyota Central R&D Labs, Inc., Nagakute, Aichi 480-1192 Japan

**Keywords:** Electrical and electronic engineering, Applied physics, Quantum mechanics, Engineering

## Abstract

Motors arise as a heart of the mobility society, and wirelessly operated motors may improve our standard of living. Wireless power transfer in the kilohertz and megahertz range has been extensively explored, finding various potential applications in consumer electronics, electric vehicles, and medical implants. However, stable operation of wirelessly powered motors remains challenging due to voltage fluctuations for motors occurring in dynamic scenarios, e.g., the rotating speed of the motors is varied. Here, we theoretically and experimentally demonstrate the operation of a motor, where the power is wirelessly transferred via coils, is robust against the rotating speed by employing the analogy with non-Hermitian parity-time (PT) symmetry. In addition, our system is robust for misalignment of the coils. Our results open up opportunities for the robust operation of motors via wireless power transfer in dynamic scenarios towards autonomous vehicles.

## Introduction

Magnetic coupling between resonators has been widely explored since the pioneering work of Ref.^1^ and has become a promising approach for efficient wireless power transfer in the kilohertz and megahertz range^2–23^. Various potential applications have been found in consumer electronics^24,25^, electric vehicles^26–28^, and medical implants^29,30^. In a system consisting of transmitting and receiving coils, there is an optimum distance between the coils for maximum power transfer, which state is called critical coupling. The performance of the power transfer degrades when the two coils are closer or away from the optimum distance, with the state being strong coupling or weak coupling, respectively. In such a strong or weak coupling state, manual tuning of impedances of coils is necessary for improving the power transfer capability^31,32^.

Non-Hermitian parity-time (PT) symmetry arising from quantum physics^33,34^ exhibits intriguing wave phenomena such as loss-induced transmission^35^, unidirectional reflectivity^36^, and chiral dynamics^37^, where the gain and loss elements have been carefully designed and balanced. Following the wide extension, the non-Hermitian PT symmetry physics was applied to wireless power transfer, where the robustness against the distance between two coils was confirmed^38,39^. Since this pioneering work, there have been significant efforts toward high efficiency and low power loss of the robust wireless power transfer against the distance^40–44^, and a charging system for a drone^45^.

**Figure 1:**
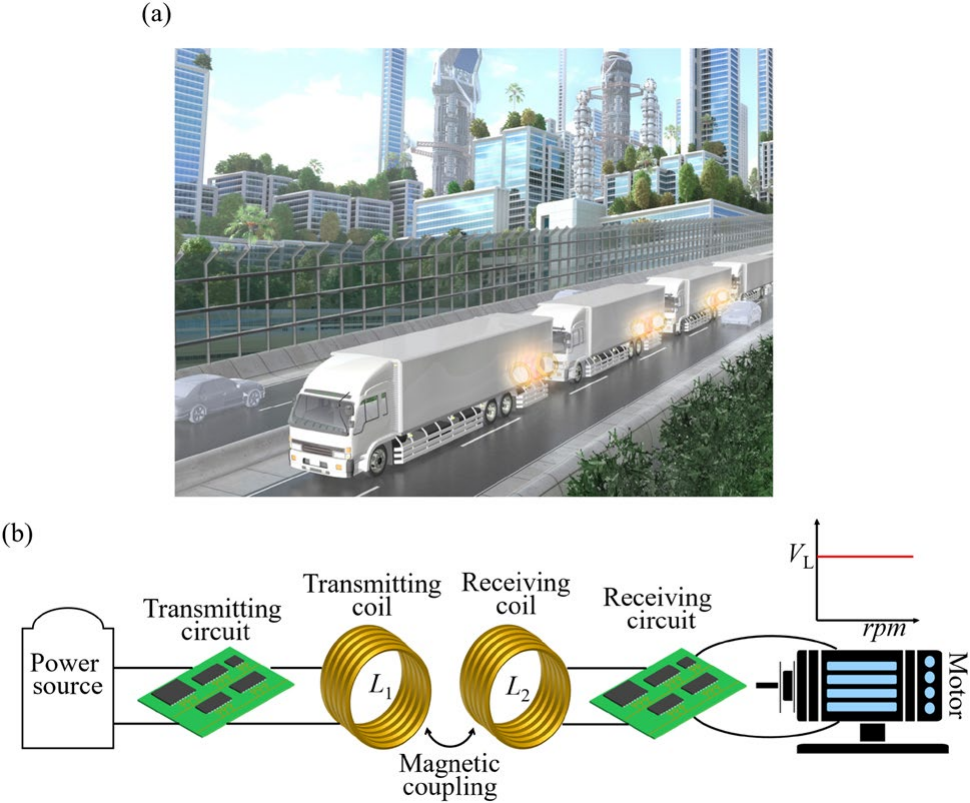
(**a**) Possible scenario of truck platooning using robust wireless power transfer among trucks. (**b**) Robust operation of a wirelessly powered motor when the rotating speed of the motor is varied.

Motors arise as a heart of the mobility society and play an important role in automobile industries, e.g., electric vehicles. For example, we suppose a platoon of electric vehicle trucks^46^, as shown in Fig. 1a. Transferring electric energy between the trucks while they are moving, may allow the increase of the cruising distance. In this context, it becomes a necessity to have robust wireless power transfer between the trucks that can be efficient against the varying distances and speeds of the trucks within the platoon. Another emerging technology for electric vehicles is in-wheel motors^47,48^, i.e., a motor is integrated into the hub of a wheel, and wireless power transfer may eliminate wire harnesses. However, in those systems, wirelessly powered motors would fail to continuously operate if the rotating speed of the motors is varied. This is because voltage fluctuations occur due to changes in the rotating speed of the motor, which may cause damage to the motors. Therefore, from a practical standpoint, ensuring the continuous wireless operation of the motor under varying rotating speeds requires robustness, which has remained a long-standing challenge. The schematic diagram illustrating the expected robust operation of a wirelessly powered motor, even when the rotating speed of the motor is varied, is shown in Fig. 1b.

In this article, we theoretically and experimentally demonstrated the wirelessly powered motor operation achieving a stable voltage that remained robust against the rotation speed of the motor. We employed the analogy with non-Hermitian PT symmetry and implemented on the wirelessly powered motor system with the half-bridge module acting as a saturable gain in the transmitting circuit and the load impedance of the motor in the receiving side as loss. We clarify the relationship between the input and the output voltage of the wireless power transfer system to perform a stable voltage for dynamic motor operations. In addition, our system is robust against misalignments of the coils; variations of the distance, angle, and offset position. Our experimental results open up opportunities of robust operation of motors via wireless power transfer in dynamic scenarios.

## Results

### Configuration and experimental setup of our wirelessly powered motor system

**Figure 2:**
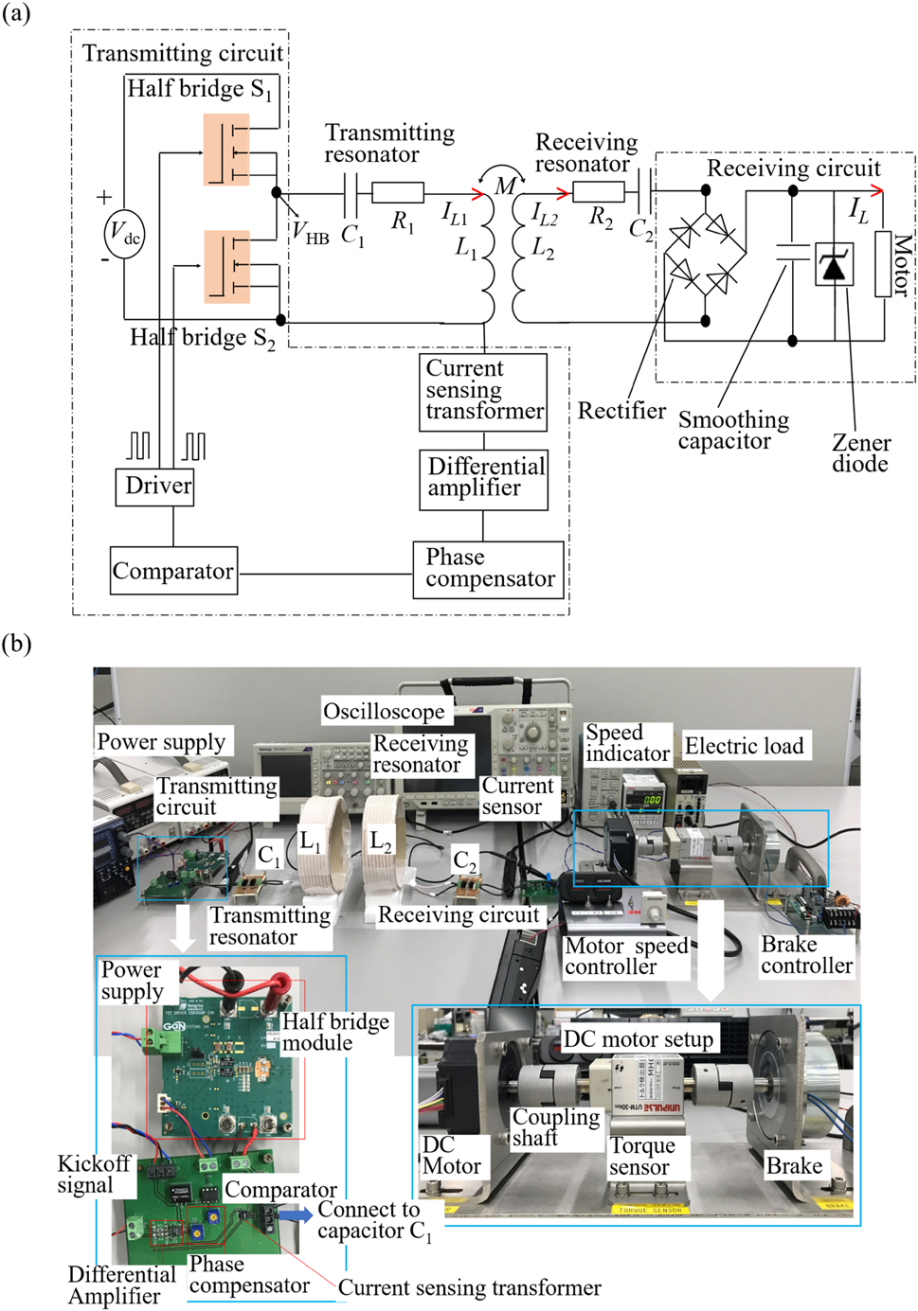
Wirelessly powered motor system using the analogy with non-Hermitian parity-time symmetry. (**a**) Circuit layout and (**b**) experimental setup. The operation power is wirelessly transferred to the dc (direct current) motor via the transmitting and receiving coils. The rotating speed of the motor was varied by the speed controller. The measurements of the rotating speed and the power were performed by the torque sensor.

We consider a wirelessly powered motor system using the analogy with non-Hermitian PT-symmetry. The motor in the receiver side is operated by wirelessly transferred power from the transmitting circuit via the transmitting and receiving resonators. Figure 2a shows the circuit diagram of our system. The transmitting and the receiving resonators are represented by capacitance ${C}_{\mathrm{1,2}}$, inductance ${L}_{\mathrm{1,2}}$, and intrinsic resistance ${R}_{\mathrm{1,2}}$, respectively, and the coupling between them is expressed by the mutual inductance $M$.

The key component for implementing non-Hermitian PT symmetry in dynamic scenarios is the non-linear negative resistance ${R}_{h}$, in which the current flows in the opposite direction for a given applied voltage^38,49^. This negative resistance ${R}_{h}$ is used as the gain source for delivering power to the transmitting resonator. The gain saturation is changed automatically as the load changes dynamically. The relationship between the gain rate ${g}_{10}$ and the input voltage of the half-bridge ${V}_{dc}$ is found in Supplementary Information. In the transmitting circuit, an input voltage ${V}_{dc}$ is given to the half-bridge module. The current sensing transformer senses the current ${I}_{L1}$ of the transmitting resonator, and then feeds to the differential amplifier, where the detected current signal is amplified. The phase compensator introduces the corresponding phase shift as the feedback signal so that the output voltage of the half-bridge circuit has the same phase as that of the current flowing in the transmitting resonator. Then the voltage signal passes to the comparator, where a rectangular driving signal is generated. Finally, the rectangular driving signal is passed to the driver so that the two identical switches (${S}_{1} \mathrm{and }{S}_{2}$) of the half bridge module are alternatively switched. (The circuit layouts of the differential amplifier and the phase compensator can be found in Supplementary.)

In the proposed WPT system, a DC (direct current) motor due to good controllability by the motor driver is selected for proving our concept. The pulse width modulation (PWM) is used for driving the DC motor. In the receiver side, the output voltage of the receiving resonator is applied to the DC motor through the full-wave rectifier, where an output AC (alternating current) voltage is converted to a DC voltage. The smoothing capacitor is inserted in parallel to the DC motor to suppress voltage ripples caused by the full-wave rectifier. The Zener diode is inserted in parallel only to avoid the damage of the motor system if in case of over-voltage flow to the DC motor, which is independent of the mechanism of our system.

Figure 2b shows the experimental setup of our system. Each of the resonators consists of a Litz wire coil (${L}_{1}$, ${L}_{2}$) and an external capacitor set (${C}_{1}$, ${C}_{2}$) on a FR4 board. The capacitor set was implemented by five capacitors of $100 \, \mathrm{pF}$, which are connected in parallel. The total capacitance is $500 \, \mathrm{pF}$. In the present study, the identical Litz wire coils [Fig. 2b] were used for both transmitting and receiving resonators. Each of the coils (${L}_{1}$, ${L}_{2}$) was fabricated by rounding up Litz wires around a cardboard tube. The strings were alternatively wounded between the windings to maintain the spacings between neighboring wires constants. Both the transmitting and receiving resonators have the same resonant frequency of $1.75 \, \mathrm{MHz}$, which is determined by the impedance spectra. Thus, each coil has an inductance of $16.65 \, \mathrm{\mu H}$.

The motor setup consists of the DC motor, the torque sensor, and the powdered brake connected through the coupling shaft. The rotating speed and the power of the DC motor are measured by the torque sensor. In the demonstration of wireless operation of the DC motor, the rotating speed of the DC motor is varied by using the motor speed controller. The brake is used to limit the rotating speed of the DC motor. We note that the current flowing through each of the transmitting coil, the receiving coil, and the DC motor is measured using the corresponding current sensor, and the current value is not used for the control of our system, but for the performance evaluation in our system.

### Analytical model: coupled model theory and circuit theory

We develop an analytic model using the coupled mode theory and circuit theory. This model allows us to provide a comprehensive explanation of our system's operation. We use the ${e}^{i\omega t}$ convention, where $\omega$ and $t$ are the frequency and time. We start by a brief review of the coupled mode theory^38,39^. The analytic model consists of two resonators, where the transmitting resonator (gain element) and the receiving resonator (loss element) are magnetically coupled. The system dynamics is described as

1 $$\frac{d}{dt}\left[\genfrac{}{}{0pt}{}{{a}_{1}}{{a}_{2}}\right]=\left[\genfrac{}{}{0pt}{}{i{\omega }_{1}+g }{-i\kappa }\genfrac{}{}{0pt}{}{-i\kappa }{i{\omega }_{2}-\gamma }\right]\left[\genfrac{}{}{0pt}{}{{a}_{1}}{{a}_{2}}\right],$$

where ${a}_{j}$ is the mode amplitude of resonator $j$, and is normalized so that ${\left|{a}_{j}\right|}^{2}$ represents the mode energy, ${\omega }_{j}$ is the resonant frequency of resonator $j$. The mode amplitude is linked to the current of the resonator as^50^,

2 $${\left|{a}_{1}\right|}^{2}=\frac{{L}_{1}{I}_{1}^{2}}{2}.$$

Resonator $1$ (transmitting resonator) has the overall gain rate of $g={g}_{10}-{\gamma }_{10}$, where ${g}_{10}$ and ${\gamma }_{10}$ are the gain rate and the intrinsic loss rate of the transmitting resonator. Similarly, resonator $2$ (receiving resonator) has the overall loss rate of $\gamma ={\gamma }_{L}+{\gamma }_{20}$, where ${\gamma }_{L}$ and ${\gamma }_{20}$ are the load of the DC motor and the intrinsic loss rate of the receiving resonator. $\kappa$ is the coupling rate between the transmitting and receiving resonators. From Eq. (1), the eigen equation is written as $\left(\omega -{\omega }_{1}\right)\left(\omega -{\omega }_{2}\right)+g\gamma -{\kappa }^{2}=0$. In our experimental setup, the two resonators have the same resonant frequency, ${\omega }_{1}={\omega }_{2}={\omega }_{0}$. Depending on the coupling rate $\kappa$ and the damping rate $\gamma$, distinctively different behaviors appear in two regimes. One is the PT symmetric phase ($\kappa \ge \gamma$, strong coupling). The saturation gain satisfying the condition of $g=\gamma$ so that the two modes have real eigen frequencies ${\omega }_{0}\pm \sqrt{{\kappa }^{2}-{\gamma }^{2}}$, and the same mode-amplitude $\left|{a}_{1}\right|=\left|{a}_{2}\right|$. The two eigen frequencies coalesce at the exceptional point, satisfying $\kappa =\gamma$. The other is the broken PT symmetric phase ($\kappa <\gamma$, weak coupling), where the two modes have the same real eigen frequency ${\omega }_{0}$, and the gain rate and the loss rate are not equal ($g\ne \gamma$).

In our analytic model, we regard the DC motor as a load. From Eqs. (1), (2), the voltage and the current of the motor are then given by^49^

3 $${V}_{L}=\sqrt{\frac{8{R}_{L}P}{{\pi }^{2}}} ,$$

4 $${I}_{L}=\sqrt{\frac{{\pi }^{2}P}{8{R}_{L}}} ,$$

5 $$P=2{\gamma }_{L}{\left|{a}_{2}\right|}^{2}=\left\{\begin{array}{cc}\frac{{\gamma }_{L}{V}_{dc}^{2}}{{L}_{1}{\pi }^{2}{\left({\gamma }_{10}+\gamma \right)}^{2}},& \kappa \ge \gamma \\ \frac{{\gamma }_{L}{\kappa }^{2}{V}_{dc}^{2}}{{L}_{1}{\pi }^{2}{\left({\kappa }^{2}+{\gamma }_{10}\gamma \right)}^{2}},& \kappa <\gamma \end{array}\right.$$

where ${R}_{L}=\frac{{V}_{L}}{{I}_{L}}$ is the load impedance of the motor. The damping rates and the coupling rate are expressed with circuit parameters; ${\gamma }_{L}={4R}_{L}/{(\pi }^{2}{L}_{2})$, ${\gamma }_{10}={R}_{1}/\left(2{L}_{1}\right)$, ${\gamma }_{20}={R}_{2}/\left(2{L}_{1}\right)$, and $\kappa ={\omega }_{0}M/2$, respectively. ${V}_{dc}$ is the input voltage of the half-bridge module. From Eqs. (3)–(5), we will calculate the voltage ${V}_{L}$, current ${I}_{L}$, and power $P$ of the motor in the PT symmetric phase as well as the broken PT symmetric phase.

We emphasize that constant voltage response is maintained in the PT symmetric phase irrespective of changes in the rotating speed (load impedance) of the motor, which can be checked in the following. From Eqs. (3) and (5), the load voltage ${V}_{L}$ of the motor can be expressed as

6 $$\left|{V}_{L}\right|=\left|{I}_{2}\right|{R}_{L}=\frac{2{V}_{dc}}{\sqrt{{L}_{1}{L}_{2}}\pi \left(\frac{{R}_{1}}{{R}_{L}}\frac{1}{{L}_{1}}+\frac{{R}_{2}}{{R}_{L}}\frac{1}{{L}_{2}}+\frac{1}{{L}_{2}}\right)} .$$

We see in Eq. (6) that voltage ${V}_{L}$ is approximately independent of ${R}_{L}$ when ${R}_{L}$> > ${R}_{1},{R}_{2}$.

### Robustness of the wirelessly powered motor system against the rotating speed

**Figure 3:**
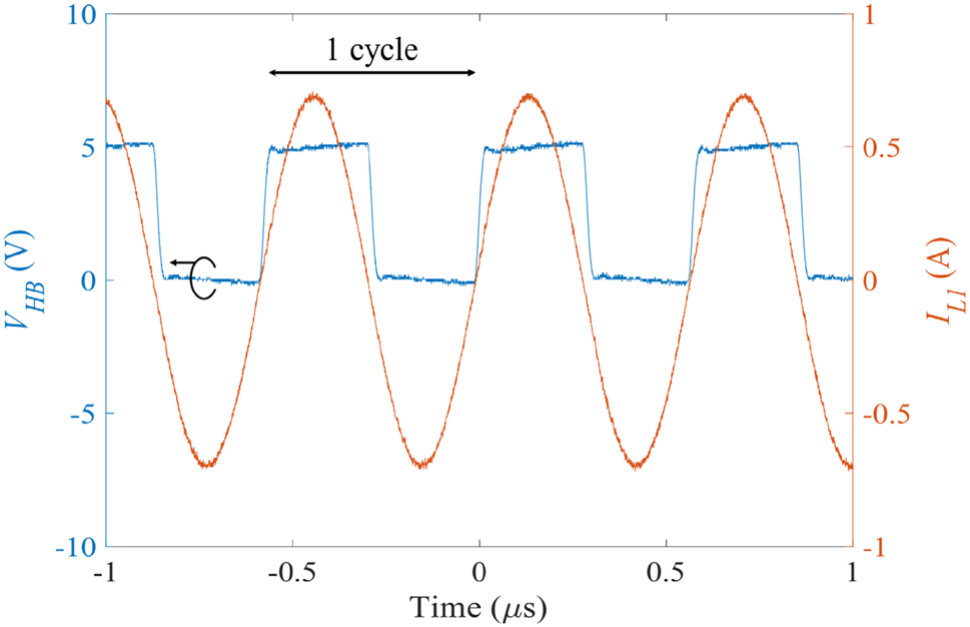
Measured time responses of the voltage *V*_HB_ induced across the half-bridge and the current *I*_L1_ flowing in the transmitting coil.

Firstly, we experimentally verified the negative resistance in the transmitting circuit where a resistive load was used instead of the DC motor. Figure 3 shows the waveforms of the half-bridge voltage ${V}_{\text{HB}}$ (blue line) and the current ${I}_{\text{L1}}$ (orange line) flowing through the transmitting coil when the resistive load was set at $5 \,\Omega$. We observe that both waveforms are in-phase with each other, which confirms that the transmitting resonator operated with the negative resistance ${R}_{h}$. In addition, we had checked that this in-phase characteristic was consistent when the resistive load was varied at $2.5 \,\Omega$, $7.5 \,\Omega$, $10 \,\Omega$, $15 \,\Omega$, and $20 \,\Omega$.

Based on the preliminary study of the transmitting circuit above, we next performed the experimental investigation of the wirelessly powered motor. The rotating speed of the motor was varied by using the PWM circuit in the PT symmetric phase, while the distance between the transmitting and receiving coils was kept constant at $d=5 \, \mathrm{cm}$. Initially, the motor was in the idle condition, and the load impedance ${R}_{L}$ of the motor was high, which was in the broken PT symmetric phase. In our experiment, the motor only operates in the PT symmetric phase avoiding the damage of the motor since high voltage may be supplied to the motor in the broken PT symmetric phase. When starting and stopping the DC motor, where the rotating speed is low, the system is in the broken PT symmetric phase. Thus, the voltage increases, which would damage the system in starting and stopping the motor. Hence, a tunable electronic resistive load (PLZ-72W, Kikusui) is connected in parallel to the motor only at starting and stopping the motor and disconnected electrically when the motor starts to operate in the PT symmetric phase, i.e., during motor operation in the PT symmetric phase, there is no loss in the tunable electronic resistive load.

**Figure 4:**
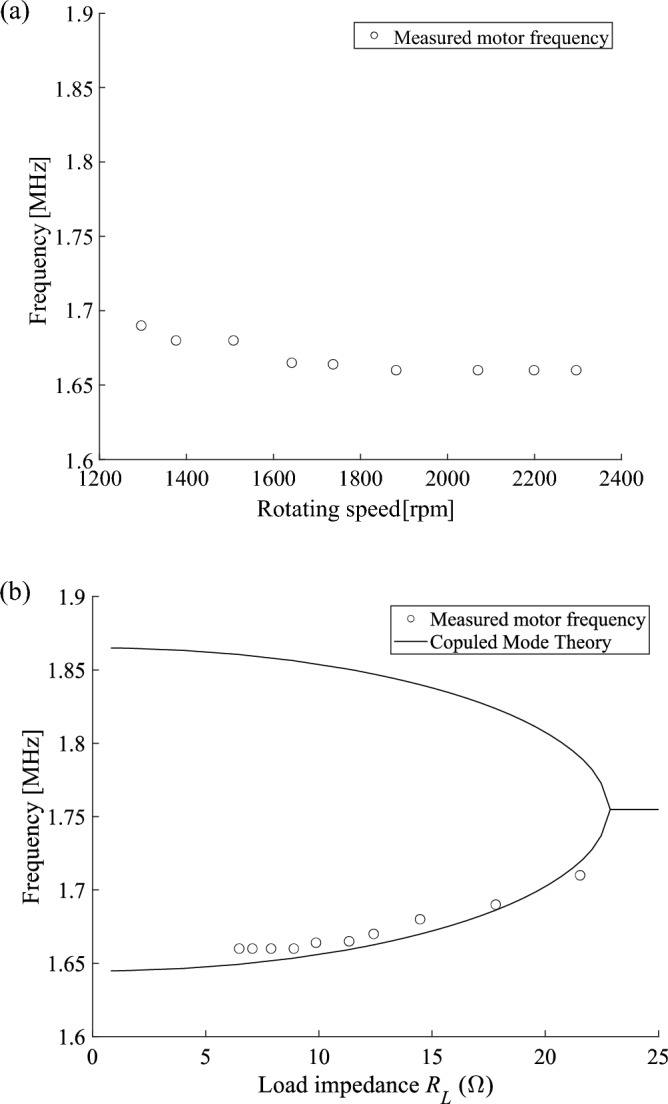
(**a**) Eigen frequencies of the system as a function of the rotating speed of the motor. (**b**) Eigen frequencies of the system as a function of load impedance ${R}_{L}$ of the motor. The measured results (symbols) of the frequencies of the motor are overlayed with the analytical results (curves) obtained by the coupled mode theory $({\omega }_{0}\pm \sqrt{{\kappa }^{2}-{\gamma }^{2}})$. Parameters are ${\omega }_{0}=1.1023\times {10}^{7}$ rad/s, $\kappa =6.9226\times {10}^{5}$ rad/s and $\gamma ={3.0395\times {10}^{4}R}_{L}$ rad/s. These values are used for other calculations unless mentioned.

Figure 4a shows the measured resonant frequency of the wirelessly operated motor system. The frequency decreases from $1.72$ to $1.66 \, \mathrm{MHz}$ as the rotating speed of the motor is varied from $1300$ to $2300 \, \mathrm{rpm}$. To validate the mechanism of our system, the analytical results are obtained by the coupled mode theory $({\omega }_{0}\pm \sqrt{{\kappa }^{2}-{\gamma }^{2}}$), where for each rotating speed of the motor, the corresponding load impedance of the motor is calculated from the current and voltage of the motor ${R}_{L}\left(=\frac{{V}_{L}}{{I}_{L}}\right)$, and are overlayed in Fig. 4b. In the PT symmetric phase, where the frequency splitting occurs, the measured frequencies (symbols) agree well with the low eigen frequency (lines) obtained by the analytic model. The operation at the low eigen frequency comes from low damping ratio, which is consistent with Ref.^51^. Therefore, we have confirmed that the operation frequency is tuned automatically.

**Figure 5:**
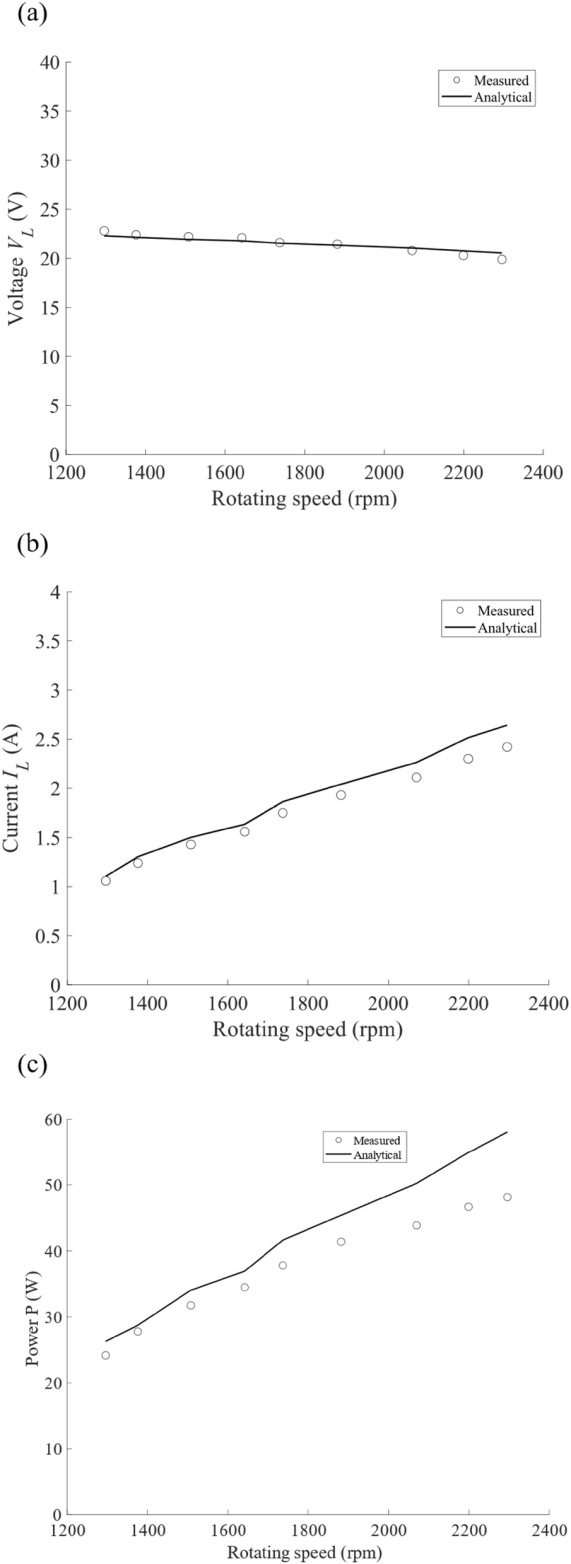
(**a**) Measured voltage *V*_*L*_ (V), (**b**) current *I*_*L*_ (A), and (**c**) power* P* (W) of the motor as a function of the rotating speed. Symbols and lines represent the measured results and analytical results obtained by the circuit model (Eqs. (3)–(5)).

In the experimental demonstration above, the voltage, current, and power of the motor were measured, and are shown in Fig. 5a–c, respectively, as the function of the rotating speed having the same range of Fig. 4a. We observe that voltage ${V}_{L}$ is almost constant around 22–23 V (Fig. 5a), as expected from the theory, even when the rotating speed of the motor is varied in the PT symmetric phase. On the other hand, current ${I}_{L}$ increases from $1.06 \, \mathrm{A}$ ($1.1 \, \mathrm{A}$) to $2.42 \, \mathrm{A}$ (2.61 $\mathrm{A}$) for the measurement results (the analytical results) in the range of 1300–2300 rpm for the rotating speed. As a result, the power $P={V}_{L}{I}_{L}$ increases from $24.16 \, \mathrm{W}$ (26.28 W) to $48.15 \, W$($57.11 \, \mathrm{W}$) for the measurement results (the analytical results), exhibiting continuous operation of the motor. The experimental results (symbols) of the voltage, current, and power agree well with the analytical results (lines) obtained from Eqs. (3)–(5). Therefore, we have verified the robustness of our system, i.e., the operation power is transferred from the transmitting circuit to the DC motor, and the DC motor is continuously operated even when the rotating speed is varied. The system efficiency of the wireless operation of the motor ranges 83–87% when the rotating speed is varied from $1300$ to $2300 \, \mathrm{rpm}$. (The motor operation in a dynamic scenario is found in the Supplemental movie.)

**Figure 6:**
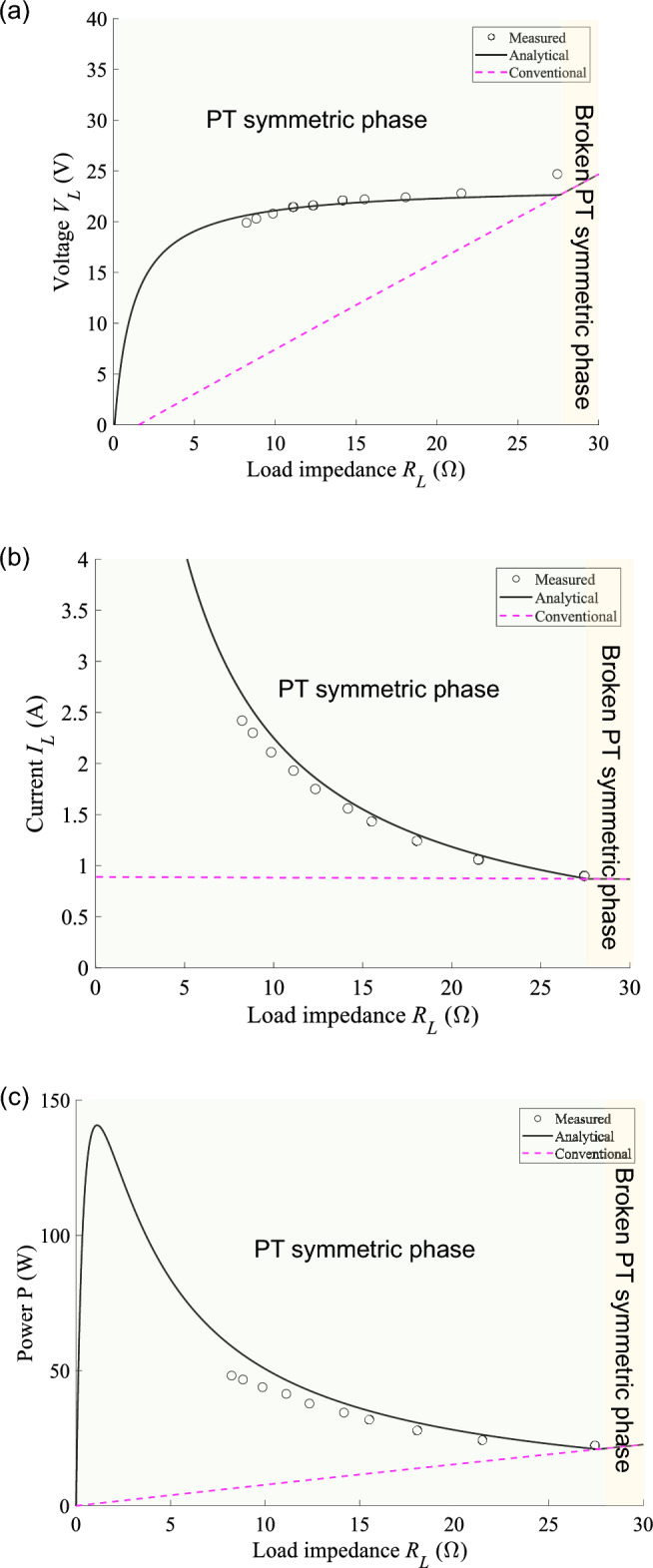
(**a**) Measured voltage *V*_*L*_ (V), (**b**) current *I*_*L*_ (A), and (**c**) power *P* (W) of the motor as a function of load impedance ${R}_{L}$ of the motor. The measured results (black symbols) are overlayed with the analytical results (black curves) obtained by the circuit model (Eqs. (3)–(5)). The pink dashed lines represent the corresponding values of a conventional wireless power transfer system.

To understand the operation principle of our system in detail, the measured voltage ${V}_{L}$, current ${I}_{L}$, and power $P$ of the motor in Fig. 5 are plotted in Fig. 6 as a function of load impedance ${R}_{L}$ of the motor ${R}_{\mathrm{L}}\left(=\frac{{V}_{\mathrm{L}}}{{I}_{\mathrm{L}}}\right)$, covering the PT symmetric and the broken PT symmetric phases. We see that our feedback scheme excellently works in the PT symmetric phase (black symbols and lines for measured and analytical results, ${R}_{L}<27\,\Omega$, with ${R}_{L}=8.2 \,\Omega$ corresponding to the rated current of the motor), contrasting with a conventional system (pink dashed lines), where the feedback scheme in the system of Fig. 2a is taken out. The circuit diagram of the conventional system^52^ can be found in Supplementary. In the conventional system with the fixed operation frequency, voltage ${V}_{L}$ and power $P$ linearly decrease with the current ${I}_{L}$ being constant, as load impedance ${R}_{L}$ decreases. In the broken PT symmetric phase, the characteristic of our system turns back to those of the conventional system ($27\,\Omega <{R}_{L}$). We plot the corresponding analytical calculation in terms of the load impedance *R*_*L*_ of the motor in Fig. 6. Thus, the experimental results (symbols) of the voltage, current, and power in Fig. 6 agree well with the analytical results (lines) obtained from Eqs. (3)–(5). Throughout the experiment, we have confirmed the negative resistance ${R}_{\mathrm{h}}$ behavior of the transmitting circuit by observing the phase match of the half-bridge voltage ${V}_{\mathrm{HB}}$ and the current ${I}_{\mathrm{L}1}$ flowing through the transmitting coil.

## Discussion

**Figure 7:**
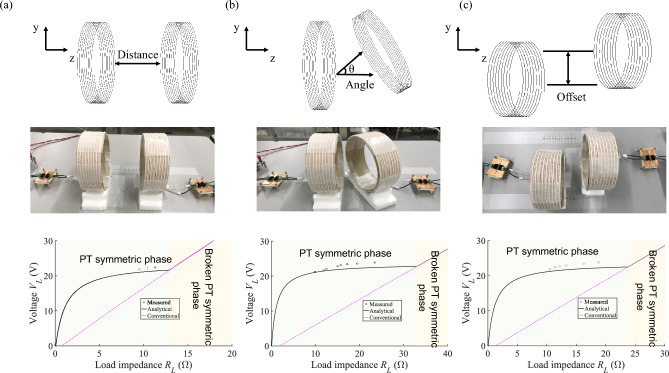
Robustness of the wirelessly powered motor system (Fig. 2) for misalignments of the coils; (**a**) distance, (**b**) angle, and (**c**) offset position. Voltages of the motor were measured with variation of the rotating speed and are plotted in the bottom panel for each case. The horizontal axis is load impedance ${R}_{L}$ so that the measured results (black symbols) of the motor are compared with the analytical results obtained from Eqs. (3)–(5) (black curves) as well as those of the conventional system (pink dashed curves).

We have so far proved that our system is robust against the rotating speed of the motor with the fixed distance between the aligned coils. Here, we experimentally show in Fig. 7 that the stable operation of the motor maintains even when misalignments of the two coils occur.

The experiment was performed for three different cases; (1) changing the distance between the transmitting and receiving coils to $d=10 \, \mathrm{cm}$ along the $z$ axis (Fig. 7a), (2) tilting the receiving coil by an angle of $30 \, \mathrm{degrees}$ from the $z$ axis (Fig. 7b), and (3) moving the receiving coil by an offset of $4 \, \mathrm{cm}$ in the $y$ direction away from the central axis of the transmitting coil [Fig. 7c]. We observe that voltages of the motor are almost constant when the rotating speed of the motor is varied in the PT symmetric phase for all the three cases. (The corresponding currents of the motor are presented in Supplementary.) The measured results show the good agreement with the analytical results, proving that our system is robust against the misalignments of the two coils as well.

We emphasize that our system operates stable without the need of impedance tuning for both transmitting and receiving coils, and communication between them in the dynamic scenario; the rotating speed of the motor and the relative position of the coils are varied. In Ref.^52^, a DC–DC converter was used in wireless power transfer. However, such DC–DC converter system would need an additional control scheme in the receiver side and the system efficiency would drop for the dynamic scenario.

In our demonstration, the motor has been continuously operated in the PT symmetric phase. Considering wide applications, here, we discuss a few possible scenarios for automobiles. On highways, automotive vehicles generally run with high rotating speed of motors. During the state, the motor in a vehicle can, in principle, be operated by the power wirelessly transferred from the neighboring vehicles, where the system is in the PT symmetric phase. When the speed of the motor reduces and the system enters the broken PT symmetric phase, the power to the vehicle may be supplied by an internal battery through an inverter for the continuous operation of the motor. On urban roads, initially the internal battery supplies the power to the motor, with the system being in the broken PT symmetric phase. When the rotating speed of the motor increases and the system reaches the PT symmetric phase, the power for the motor operation can be switched from the internal battery to the neighboring vehicles and vice-versa. Our feedback scheme is not limited to the operation of DC motors, and may be used for other types of motors, e.g., AC motors such as induction motors and synchronous motors for high power. In the context of automotive applications, it would be interesting to explore the potential of regenerative braking in our system.

In conclusion, we have experimentally demonstrated that a DC motor continuously operates in the receiver side by the operation power wirelessly transferred from the transmitting circuit in dynamic scenarios; the rotating speed of the motor is varied, and misalignments of the coils occur. The implementation of non-Hermitian PT symmetry in our system allowed the stable and efficient operation of the motor without manual tuning and no additional control scheme is required. Our results open up opportunities of robust operation of motors via wireless power transfer in dynamic scenarios towards autonomous vehicles. Future works include exploring the possibilities of other types of motors for our system and the motor operation in the broken PT symmetric phase.

## Methods

The transmitting and receiving circuits were implemented on FR4 boards. The transmitting circuit was implemented with a half-bridge module (GS61008P), current sensing transformer (CU8965), differential amplifier, phase compensator, and comparator (AD8561ANZ). The current sensing transformer is connected to the transmitting coil to pick up the current. The differential amplifier consisted of an operational amplifier (LT1223) and four resistors. The phase compensator was achieved by a low pass filter. Voltages of $+50 V$, $\pm 12 V$, and $+5 V$ were supplied to the half-bridge module, the differential amplifier, and the comparator, respectively, from DC voltage sources (ZX-1600HA, PW36-15AD, and PW18-2ATP). Oscilloscopes MSO4104B-L (Tektronix) and TDS2024C (Tektronix), and current sensors TCP0030 (Tektronix), TCP0030A (Tektronix), and TCPA300 (Tektronix) were used in our experiments.

In the receiver side, we have used a full wave rectifier composed of four diodes (RB075BGE40S), smoothing capacitor (ECA-2AM101), Zener diode (1N5361BG), and DC motor (BLHM450K-A). The micro-powdered brake (OPC40N) was coupled to the DC motor and controlled by using a constant current controller (CTA1100). In our demonstration, we varied the rotating speed of the DC motor by using the PWM circuit (BLH2D50-K).

### Supplementary Information

Supplementary Information 1.

Supplementary Video 1.

## Data Availability

The data that support the present study are available from the corresponding author upon reasonable request.
