# Supplementary material for: Wirelessly powered motor operation in dynamic scenarios using non-Hermitian parity-time symmetry
Source: Sci Rep. 2023 Dec 6;13:21492. doi: 10.1038/s41598-023-47842-x (PMC10700535; doi:10.1038/s41598-023-47842-x)
Supplement: Supplementary file 1 — Supplementary Information 1. [file 41598_2023_47842_MOESM1_ESM.pdf]

Supplementary Information: Wirelessly powered motor operation in dynamic scenarios using non-Hermitian parity-time symmetry

Shrinathan Esaki Muthu Pandara Kone, Kenichi Yatsugi, Hideo Iizuka

Toyota Central R&D Labs., Inc., Nagakute, Aichi 480-1192, Japan

S1. Circuit layouts of the differential amplifier and the phase compensator

The differential amplifier consists of an operational amplifier (LT1223) and four resistors with resistances being $R_1 = R_2 = 1\ \mathrm{k\Omega}$ and $R_3 = R_4 = 2.1\ \mathrm{k\Omega}$ [Fig. S1(a)] so that it has a gain of $A = 2$. Voltages of $\pm 12\ \mathrm{V}$ are applied to the operational amplifier.

The phase compensator consists of two variable resistors with resistances of R_{V1} and R_{V2}, and a capacitor with a capacitance of $470\ \mathrm{pF}$ [Fig. S1(b)]. The resistances R_{V1} and R_{V2} have variable ranges of $200\ \Omega$ and $1\ \mathrm{k\Omega}$, respectively.

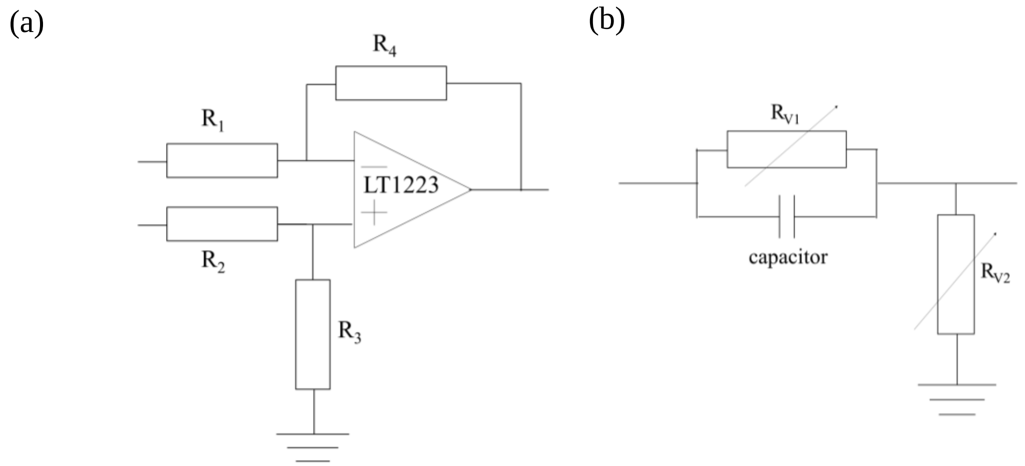

Fig. S1. Circuit layouts of (a) the differential amplifier and (b) the phase compensator.

S2. Parameters of fabricated coils

Table S1 shows the parameters of the fabricated coils for the transmitting and receiving resonators.

Table S1. Parameters of fabricated coils.

Diameter of wires	1.6 mm
Number of turns of wires	9
Diameter of strings	4 mm
Diameter of coils	16 cm

S3. Circuit diagram of the conventional system

In the main text, a conventional system is presented, where the control scheme in the system of Fig. 2(a) is taken out. The circuit diagram of the conventional system is shown in Figure S2. The inverter and the DC/DC converter are incorporated into the conventional system.

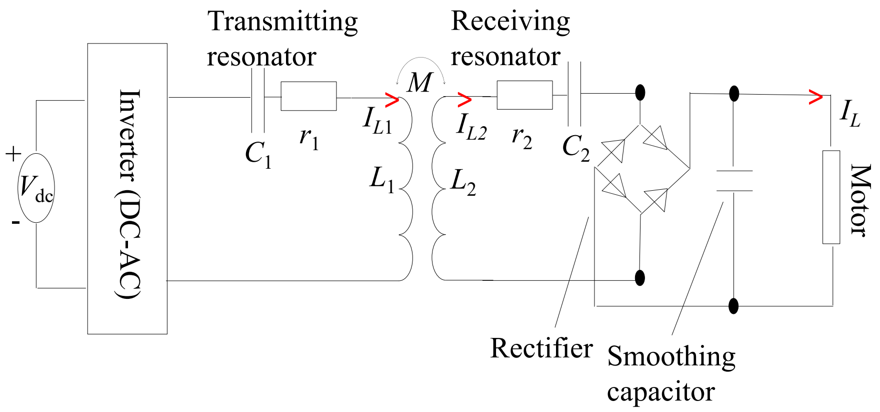

Fig. S2. Circuit layout of the conventional wireless power transfer system.

We derive the power, voltage, and current of the system of Fig. S2 from the circuit diagram above and the coupled mode theory formalism. Voltage V_2 of the receiving coil is expressed with voltage V_1 of the transmitting coil and load impedance R_L [1],

$$V_2 = \frac{j\omega_0 k L_1 R_L}{r_1(r_2 + R_L) + (\omega_0 k L_1)^2} V_1, \quad \text{(S1)}$$

where the resonant frequency ω_0 of the transmitting resonator is given as

$$\omega_0 = \frac{1}{\sqrt{L_{1,2} C_{1,2}}}. \quad \text{(S2)}$$

r_1 and r_2 are the intrinsic losses of the transmitting and receiving resonators, respectively, and k is the coupling coefficient between the resonators. Voltage V_1 of the transmitting coil can be calculated using input voltage V_{dc} of the half-bridge module.

$$V_1 = \frac{\sqrt{2}}{\pi} V_{\mathrm{dc}}. \quad \text{(S3)}$$

Then current I_2 flowing through the receiving coil is given as[1]

$$I_2 = \frac{j\omega_0 k L_1}{r_1(r_2 + R_L) + (\omega_0 k L_1)^2} V_1. \quad \text{(S4)}$$

Using Eqs. (S3) and (S4), the power flowing load impedance R_L is written as

$$P = |I_2|^2 R_L = \frac{(\omega_0 k L_1)^2 R_L}{[r_1(r_2 + R_L) + (\omega_0 k L_1)^2]^2} \left(\frac{\sqrt{2}}{\pi} V_{dc}\right)^2. \quad \text{(S5)}$$

Using Eq. (S5), voltage V_L and current I_L of load impedance R_L are expressed

$$V_L = \sqrt{P R_L} = \frac{\omega_0 k L_1 R_L}{r_1(r_2 + R_L) + (\omega_0 k L_1)^2} \left(\frac{\sqrt{2}}{\pi} V_{dc}\right), \quad \text{(S6)}$$

$$I_L = \sqrt{P / R_L} = \frac{\omega_0 k L_1}{r_1(r_2 + R_L) + (\omega_0 k L_1)^2} \left(\frac{\sqrt{2}}{\pi} V_{dc}\right). \quad \text{(S7)}$$

S4. Relationship between the gain rate and half-bridge input voltage

The relationship between the gain rate g_{10} and the current flowing through the transmitting resonator I_{L1} is discussed below. The relationship between the gain rate g_{10} and negative resistance R_h is shown as [2],

$$g_{10} = \frac{R_\mathrm{h}}{2L_1}. \quad \text{(S8)}$$

Since the half-bridge circuit generates a square wave voltage, its fundamental wave component is $\sqrt{2}V_{dc}$[1]. The negative resistance R_h is given from Ohm's law

$$R_\mathrm{h} = \frac{\sqrt{2}V_\mathrm{dc}/\pi}{I_{L1}}. \quad \text{(S9)}$$

Substituting Eq. (S9) into Eq. (S8), we have

$$g_{10} = \frac{\sqrt{2}V_\mathrm{dc}/\pi}{2L_1 I_{L1}}. \quad \text{(S10)}$$

Thus, the gain value decreases as the current flowing through the transmitting resonator increases. The current depends on the load impedance changes.

S5. Currents of the motor for the misalignments of the coils in Fig. 7

Figure S3(a)-(c) shows the corresponding currents of the motor for the misalignments of the coils; (a) distance, (b) angle, and (c) offset position in Fig. 7. The currents increase as the rotating speed of the motor decreases in the PT symmetric phase for all the three cases. The experiment results (symbols) agree well with the analytical results (lines).

(a) (b) (c)

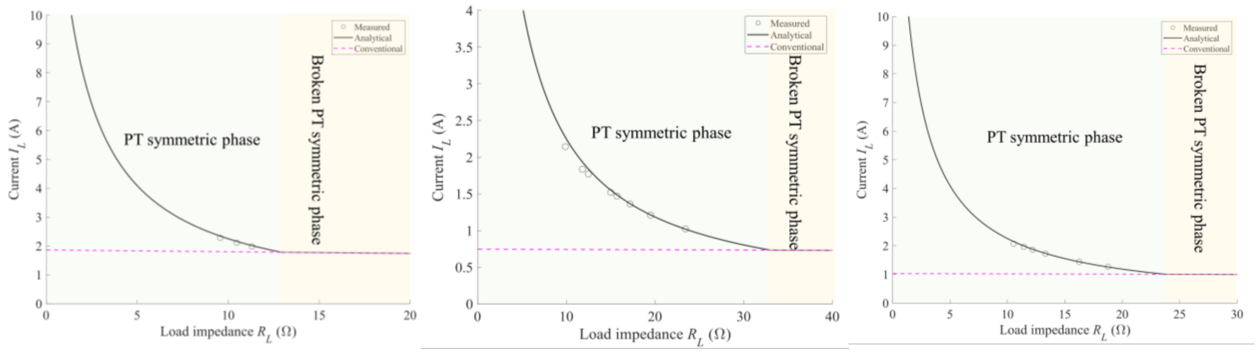

Fig. S3. Corresponding currents of the motor for the misalignments of the coils; (a) distance, (b) angle, and (c) offset position in Fig. 7. The horizontal axis is the load impedance so that the measured results (black symbols) of the motor are compared with the analytical results obtained from Eqs. (3)-(5) (black curves) as well as those of the conventional system (pink dashed curves).

Reference

1 Imura, T. *Wireless Power Transfer: Using Magnetic and Electric Resonance Coupling Techniques*. (Springer; 1st ed. 2020 edition, 2021).

2 Rong, C., Zhang, B., Wei, Z., Wu, L. & Shu, X. A Wireless Power Transfer System for Spinal Cord Stimulation Based on Generalized Parity–Time Symmetry Condition. *IEEE Transactions on Industry Applications* **58**, 1330-1339, doi:10.1109/tia.2021.3090751 (2022).
